# Phylogenetic and Mutation Analysis of Hemagglutinin Gene from Highly Pathogenic Avian Influenza Virus H5 Clade 2.3.4.4b in South America

**DOI:** 10.3390/v17070924

**Published:** 2025-06-28

**Authors:** Alfredo Bruno, Domenica de Mora, Miguel Angel Garcia-Bereguiain, Juan Cristina

**Affiliations:** 1 Instituto Nacional de Salud Pública e Investigación, Guayaquil, Ecuador; 2 Universidad Agraria del Ecuador, Guayaquil, Ecuador; 3 One Health Research Group, Universidad de Las Américas, Quito 170516, Ecuador; 4 Laboratorio de Virología Molecular, Centro de Investigaciones Nucleares, Facultad de Ciencias, Universidad de la República, Igua 4225, Montevideo 11400, Uruguay

**Keywords:** avian influenza virus, clade 2.3.4.4b, migratory birds, viral evolution, HPAIV, South America

## Abstract

The Highly Pathogenic Avian Influenza Virus (HPAIV) H5 clade 2.3.4.4b has caused severe outbreaks in domestic and wild birds worldwide since its emergence in 2014, and especially since 2020, with outbreaks in Europe and North America. The introduction of the virus into South America was reported for the first time in Colombia in October 2022, followed by outbreaks in other South American countries affecting poultry, wild birds, mammals, and humans. In this study, a phylogenetic and mutation analysis of the hemagglutinin (HA) gene of HPAIV H5N1 2.3.4.4b viruses isolated in South America was performed to analyze its evolution and its transmission and zoonotic potential. The analysis shows an increase in the viral effective population size between April and June 2022, which was followed by multiple outbreaks of HPAIV H5N1 clade 2.3.4.4b in South America. Moreover, the virus variants evolved from a recent common ancestor estimated to have existed in June 2017. The mean rate of evolution of the HA gene was 6.95 × 10^−3^ substitutions per site per year, and the sequence analysis of HA identified a mutation (D171N) located at antibody binding sites and viral oligomerization interfaces, with implications for immune response evasion and new host species infection. Additionally, viral strains from South America share the substitutions L104M, T156A, P181S, and V210A, compared to the vaccine strain A/chicken/Ghana/AVL763/2021. Understanding the dynamics of viral evolution and transmission is essential for effective prevention strategies to mitigate future outbreaks.

## 1. Introduction

Avian influenza viruses (AIVs) represent a considerable challenge to public health systems worldwide due to their widespread circulation and significant mortality rates [1]. AIVs belong to the family *Orthomyxoviridae*, and their genome consists of eight segments of negative polarity that encode for at least 11 different proteins, including hemagglutinin (HA) and neuraminidase (NA) glycoproteins. Avian HA and NA are classified into 16 and 9 subtypes, respectively [1].

AIVs can be divided into two different groups based on their pathogenicity: highly pathogenic avian influenza viruses (HPAIVs) and low pathogenic avian influenza viruses (LPAIVs) [2]. Among the various HPAIV types, the H5N1 virus is regarded as the most pathogenic, with a high mortality rate in birds and also in humans. In recent years, frequent outbreaks of HPAIV H5N1 have occurred worldwide, raising concerns about its pandemic potential affecting humans [3].

HPAIV H5N1 can be divided into 10 primary clades (designated as 0 to 9) and numerous secondary and diverse sub clades [4]. H5 viruses have undergone several intercontinental waves, and the most recent one is currently ongoing and includes the clade 2.3.4.4b [5,6]. Since 2020, clade 2.3.4.4b has spread from Asia to Europe, Africa, and North America through migratory birds, causing an unprecedented number of deaths in poultry and also affecting a wide range of wild birds and mammal species [7,8,9,10]. This clade was identified in North America and Canada in late 2021, reaching Colombia, Venezuela, and Ecuador by the end of 2022, and spreading all over South America and to Antarctica since then [11,12,13].

Regarding the capacity of HPAIV to infect a wide range of hosts, special attention is given to mutations in the HA gene [14]. HA is the viral protein responsible for binding to viral receptors (α2→3 sialylated glycans), and the hallmark of human-adapted HA subtypes is a “quantitative switch” in their binding preference to human receptors (α2→6 sialylated glycan) [15]. The receptor binding site (RBS) within HA has conserved structural features, including the 130- and 220-loops and the 190-helix. Mutations at this site should be closely monitored, as they can lead to a shift in binding preference toward human receptors (SA-α2,6) [16,17].

The aim of this study is to perform a mutation and phylogenetic analysis of the HA gene for HPAIV H5N1 clade 2.3.4.4b in South America to gain insights into changes in transmission and zoonotic potential.

## 2. Materials and Methods

### 2.1. Sequences

A total of 157 full-length sequences from the HA gene of HPAIV H5N1 2.3.4.4b were obtained from the Global Initiative on Sharing Avian Influenza Data (GISAID) database in the period between December 2019 and September 2023. For accession numbers, country of origin, and date of isolation, see Supplementary Material Table S1.

We did not analyze HPAIV whole genome sequences or multiple genes, but only HA sequences. Our mutation and phylogenetic analysis is based in HA sequences for the following reasons: (1) this region defines the name of the different clades used to classify the virus; (2) HA is a very relevant protein involved in host interaction, and its evolution is linked to changes in host/virus interaction and vaccine effectiveness; (3) there are more HA sequences in GISAID than whole genome sequences, so an extensive phylogenetic analysis, such as the one presented here, is only possible with HA sequences.

### 2.2. Sequence Alignment

Sequences were aligned using the MAFFT version 7 program [18].

### 2.3. Bayesian Markov Chain Monte Carlo Analysis

To investigate the evolutionary patterns of HPAIV H5N1 2.3.4.4b variants circulating in the American region, a Bayesian Markov Chain Monte Carlo (MCMC) approach was used as implemented in the BEAST package v2.5.2 [19]. First, the evolutionary model that best fit the sequence dataset was determined using software from the IQ-TREE(version 2.4.0) program [20]. Bayesian information criterion (BIC), Akaike information criterion (AIC), and the log of the likelihood (LnL) indicated that the HKY + ⌈ model was the most suitable model. Both strict and relaxed molecular clock models were used to test different dynamic models (constant population size, exponential population growth, expansion population growth, logistic population growth, and Bayesian Skyline). Statistical uncertainty in the data was reflected by the 95% highest probability density (HPD) values. Results were examined using the TRACER v1.6 program (available from http://beast.bio.ed.ac.uk/Tracer; accessed on 15 March 2025). Convergence was assessed by effective sample sizes (ESS) above 200. Models were compared by AICM from the likelihood output of each of the models using the TRACER v1.6 program. Lower AICM values indicate better model fit. The Bayesian Skyline model was the best model to analyze the data. Maximum clade credibility trees were generated by means of the use of the Tree Annotator program from the BEAST package. Visualization of the annotated trees was performed using the FigTree program v1.4.2 (available at: http://tree.bio.ed.ac.uk, accessed on 29 March 2025). A Bayesian Skyline was constructed using TRACER 1.6 software.

### 2.4. Substitution Analysis in the HA Protein of HPAIV H5N1

In order to identify key substitutions in the HA protein of HPAIV H5N1 that may be related to phenotypic changes or special epidemiological relevance, HA nucleotide sequences from HPAIV H5N1 2.3.4.4b isolated in South America were translated in silico using the MEGA program version 11 [21] and compared with the WHO-recommended vaccine strain A/chicken/Ghana/AVL763/2021 (clade 2.3.4.4b). The strain A/chicken/Ghana/AVL763/2021 was selected because it is the most updated vaccine candidate recommended by the WHO to prevent the spread of the current HPAIV H5N1 panzootic to a potential human epidemic.

### 2.5. Prediction of N-Linked Glycosylation Sites in the HA Protein

Potential N-linked glycosylation sites in the HA protein were predicted using the NetNGlyc 1.0 Server [22]. The NetNglyc server predicts N-Glycosylation sites in proteins using artificial neural networks that examine the sequence context of Asn-Xaa-Ser/Thr sequons. A threshold value of >0.5 average potential score was set to predict glycosylated sites.

### 2.6. Mapping of Amino Acid Substitutions in a 3D Structure of HPAIV H5N1 HA Protein

Amino acid substitutions found in the globular head domain (HA1) of HA proteins from HPAIV H5N1 2.3.4.4b strains isolated in South America were mapped in the 3D structure HA protein from HPIAV A/gyrfalcon/Washington/41088-6/2014 (H5N8) (2.3.4.4 clade), available at the Protein Data Bank (PDB) under accession number 5HUF. Visualization was performed using Jmol-14.0.4 software (available at: http://www.jmol.org/, accessed on 29 March 2025).

### 2.7. Tridimensional Models of Receptor Binding Sites

The 3D model of the receptor binding site of the recommended vaccine strain A/chicken/Ghana/AVL763/2021 (clade 2.3.4.4b) was obtained using Phyre 2 [23]. The 3D model obtained for the recommended HPAIV H5N1 vaccine strain is available in Figure S1. A total of 100% of the RBS sequences were modeled with 100.0% confidence. The strain A/chicken/Ghana/AVL763/2021 was selected because it is the most updated vaccine candidate recommended by WHO to prevent the spread of the current HPAIV H5N1 panzootic to a potential human epidemic.

## 3. Results

### 3.1. Phylogenetic and Population Analysis of HPAIV H5N1 2.3.4.4b Strains from South America Based on HA Sequences

A phylogenetic analysis using 157 available and comparable full-length HA sequences from HPAIV H5N1 2.3.4.4b strains isolated elsewhere was performed using a Bayesian MCMC approach [19] (the complete list of viral sequences included in these analyses is detailed in Supplementary Material Table S1). The parameters for the Bayesian analysis are shown in Table 1. The value obtained for the time of the most common recent ancestor (tMRCA) for the South American HPAIV H5N1 2.3.4.4b was estimated in June 2017. A mean rate of evolution of 6.95 × 10^−3^ substitutions per site per year (s/s/y) was found for the HA gene sequences included in these studies.

**Figure 1:**
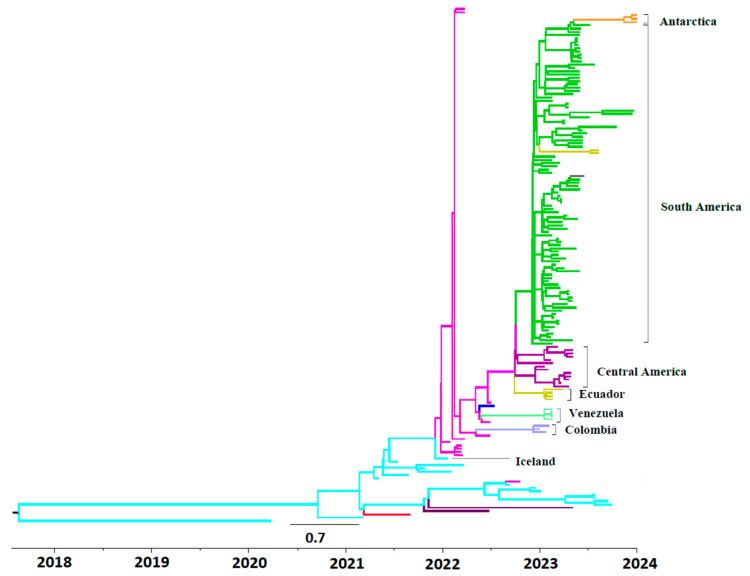
Bayesian MCMC phylogenetic tree analysis of 2.3.4.4b H5N1 HPIA strains circulating in South America. A maximum clade credibility tree obtained using the HKY + γ model, a relaxed molecular clock, and the Bayesian Skyline population model is shown. The tree is rooted to the Most Recent Common Ancestor (MRCA). Time to the MRCA is shown in years at the bottom of the figure. The bar at the bottom of the tree denotes time in years. Strains in the tree are indicated by different colors according to their geographic location of isolation, which is indicated next to the clade. The vaccine strain recommended by the WHO A/chicken/Ghana/AVL763/2021 is shown in red. Clades of strains isolated in Europe are shown in cyan, while clades of strains isolated in North America are shown in fuchsia. Strains isolated in Asia are shown in brown. Human strains A/Colorado/18/2022 and A/Chile/25945/2023 are shown in blue and gray, respectively.

The maximum clade credibility tree for HA sequences from HPIAV H5N1 2.3.4.4b strains from South America is shown in Figure 1. The first HPIAV H5N1 2.3.4.4b sub clade in South America was found in Colombia, dated in 16 September 2022 (95% HPD interval from 14 to 27 September 2022), followed by other sub clades including HPAIV H5N1 strains isolated in Venezuela and Ecuador, and also another sub clade including strains isolated in Mexico and Central America (Figure 1). Additionally, a large HPAIV H5N1 sub clade was found, including sequences isolated in Peru, Chile, Argentina, and Uruguay, the extreme south of South America (Araucaria, Chile), and Antarctica.

**Figure 2:**
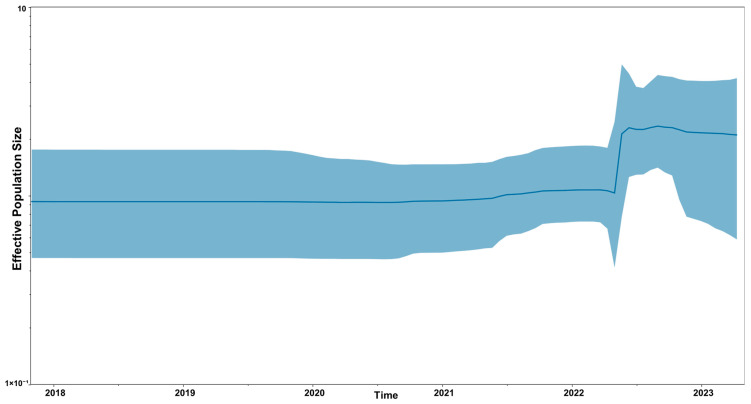
Bayesian Skyline plot depicting the population history of HPIA H5N1 strains isolated in South America. The thick solid black line represents the median estimate, and the blue area shows the 95% highest probability density (HPD) values.

A Bayesian Skyline plot for the population history of HPAIV H5N1 2.3.4.4b strains from South America is shown in Figure 2. A sharp increase in viral effective population size was found by mid-2022.

### 3.2. Mutation Analysis of the HA Gene of HPAIV H5N1 2.3.4.4b Strains from South America

**Figure 3:**
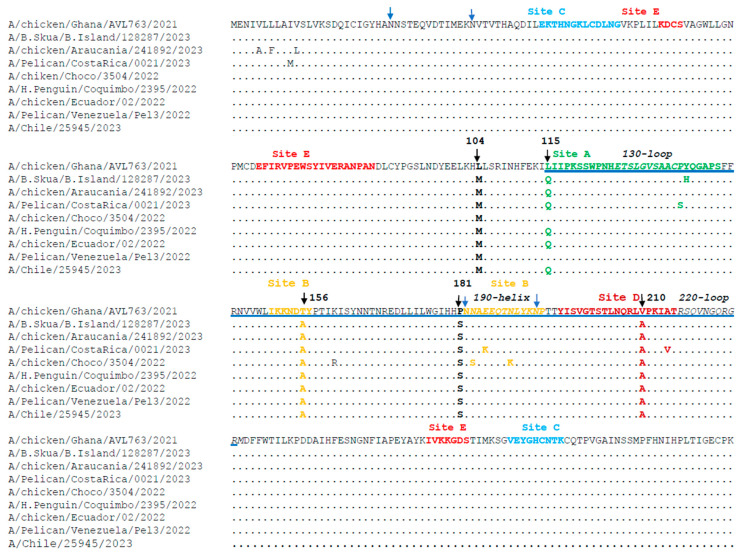
Substitutions found in the HA1 of the HA protein of H5N1 strains. The amino acid sequence of the recommended vaccine strain A/chicken/Ghana/AVL763/2021 is shown. Identity to this strain is indicated by a dot. Numbers at the top of the sequence denote amino acid positions. The five antigenic regions of clade 2.3.4.4b are highlighted in different colors and indicated on top of the alignment. Amino acids containing the receptor binding site (RBS) are shown underlined in blue. The structural motifs 130-loop, 190-helix, and 220-loop are shown in italics. Glycosylation sites are shown by blue arrows. Positions of substitutions found in most HPIA H5N1 strains isolated in South America are shown by black arrows.

The sequences of the globular head domain (HA1) of the HA gene from 106 H5N1 2.3.4.4b strains from South America were translated in silico and compared with sequences from the WHO-recommended vaccine A/chiken/Ghana/AVL763/2021. As detailed in Section 2, the strain A/chicken/Ghana/AVL763/2021 was selected because it is the most updated vaccine candidate recommended by the WHO to prevent the spread of the current HPAIV H5N1 panzootic to a potential human epidemic. Some examples of these comparisons are shown in Figure 3. The sequence analysis of HA identified a mutation (D171N) located at antibody binding sites and viral oligomerization interfaces, with implications for immune response evasion and new host species infection. Moreover, all the 2.3.4.4b strains from South America (including the strain A/Chile/25945/2023 from a human case in Chile) share the substitutions L104M, T156A, P181S, and V210A (Figure 3). The substitution L115Q was present in 90% of the viral strains. The substitution Q226L associated with mammalian transmission was not found in the sequences analyzed in our study.

**Figure 4:**
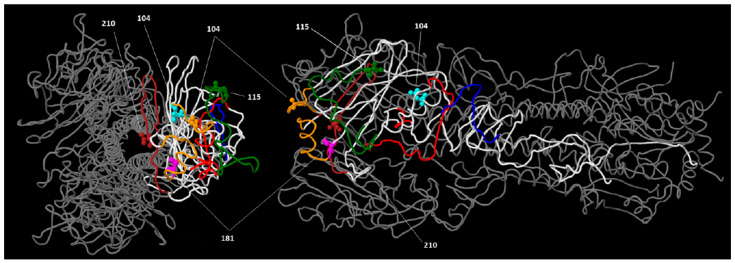
Mapping of amino acid substitutions found in the HA1 of HPIA H5N1 strains isolated in South America. The trimeric 3D structure of HA of strain A/gyrfalcon/Washington/41088-6/2014 (clade 2.3.4.4) is shown (PDB accession number 5HUF). Substitutions found in H5N1 strains isolated in South America are indicated in space-filling representation, and their position in HA1 is indicated in one molecule of the trimer. Antigenic regions are shown in one of the molecules in the same colors as Figure 3. Two views of the molecule, rotated on the *x*-axis, are shown.

These substitutions were mapped in the 3D HA structure of A/gyrfalcon/Washington/41088-6/2014 (clade 2.3.4.4), as detailed in Figure 4. Most of the substitutions map at the top of the HA molecule, affecting at least two different antigenic sites. This information is important to develop neutralizing antibodies and effective vaccines [24].

**Figure 5:**
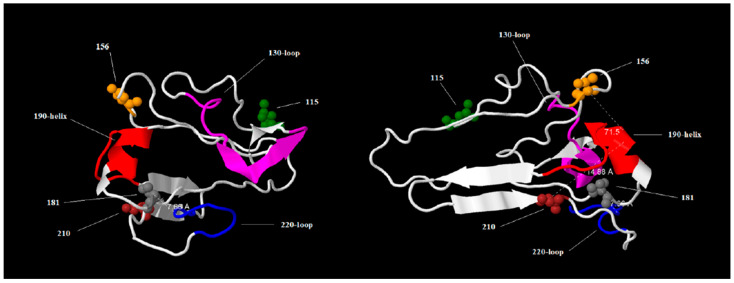
3D receptor binding site (RBS) model. The 3D model of the RBS of the recommended vaccine strain A/chicken/Ghana/AVL763/2021 is shown in white. The structural motifs 130-loop, 190-helix, and 220-loop are shown in fuchsia, red, and blue, respectively. Substitutions found in HPIA H5N1 strains isolated in South America are shown in space-filling representation in green, orange, gray, and brown, and their positions are indicated by numbers. Two views of the molecule, rotated on the *x*-axis, are shown. Dotted lines show distance in A.

Previous studies have demonstrated that monoclonal antibodies (mAbs) epitopes that specifically target RBS have the potential to directly inhibit viral binding [25]. For this reason, the substitutions found in HPIAV H5N1 strains isolated in South America were studied in the context of RBS molecular features. Due to the fact that the 3D structure of recommended vaccine strain A/chicken/Ghana/AVL763/2021 is unknown, the RBS of this strain was modeled using Phyre 2 software(version 2.2) [23] (Figure S1), and this model was used to map the substitutions found in the HPAIV H5N1 strains isolated in South America (Figure 5). The results of these studies revealed that substitutions in RBS shared by all HPAIV H5N1 isolated in South America do not map in the relevant structure motifs (130-loop, 190-helix, and 220-loop). Although the substitutions found do not map to relevant motifs, we cannot rule out that these peripheral mutations may alter HA conformational dynamics, affecting receptor avidity or antigenicity.

## 4. Discussion

The phylogenetic analysis conducted in this study revealed a complex pattern of introduction and spread of the HPAIV H5N1 2.3.4.4b in South America. It has been reported that this virus arrived in North America via migratory birds from northwest Europe, and from there it spread through South America [5,11]. This finding aligns with previous research indicating that wild birds play a crucial role in the intercontinental spread of HPAIV [9].

Bayesian coalescent analysis of HPAIV populations circulating in South America revealed an increase in the effective size of the viral population between the months of April and June 2022, associated with the increased transmission of HPAIV H5N1 2.3.4.4b in South America.

The mutation analysis of the HA gene for HPAIV H5N1 isolates from South America identified a significant one (D171N), located at antibody recognition sites and viral oligomerization interfaces, and involved in the binding of small ligands. This mutation could potentially impact the virus’s ability to infect different hosts and evade the immune response. However, further studies are needed to confirm these effects. On the other hand, the mutation Q226L (involved in mammalian transmission and described for HPAIV H5N1 isolated from North America) has not been found in the sequences analyzed for South America in the current study.

The findings of this study have important implications for the control and prevention of the H5N1 clade 2.3.4.4b influenza virus. The identification of the routes of introduction and spread of the virus can inform surveillance strategies and interventions to prevent future outbreaks. The detection of significant mutations can guide the development of vaccines and antiviral drugs. Continued surveillance of HPAIVs in South America is recommended, including specific actions like sentinel surveillance of poultry farms and wild bird and aquatic mammal populations, and also to enhance regional capacities for whole genome sequencing of HPAIV.

Our study has several limitations that we want to acknowledge. The analysis was based on available sequences, which may not represent all circulating viruses. We are aware of the potential geographical bias due to the fact that most of the 157 sequences included in the study are from Colombia, Peru, and Chile, with fewer sequences coming from other countries like Ecuador, Bolivia, or Brazil. However, we cannot solve this issue as we were taking sequences from the GISAID database. This potential geographical bias is based on the differences in sequencing efforts across South American countries. Additionally, the study focuses solely on the HA gene, not addressing potential reassortment and mutation events in other segments like NA and PB2 that could influence pathogenicity and host adaptation. Additionally, the study did not investigate the impact of the identified mutations on the virulence and transmissibility of the virus; experimental studies are needed to determine the effects of the identified mutations on the biological properties of the virus.

In conclusion, this study contributes to our understanding of the evolution and spread of the HPAIV H5N1 2.3.4.4b in South America and highlights the need for sentinel surveillance of poultry and wild bird and mammal populations, and to reinforce genomic surveillance.

## Figures and Tables

**Table 1:** Bayesian coalescent inference of HPIA H5N1 strains.

Group ^a^	Parameter	Value ^b^	HPD ^c^	ESS ^d^
HA full-length sequences	Posterior	−5557.58	5593.94 to −5520.62	2086.00
	Prior	−232.58	−268.24 to −195.78	1497.60
	Tree likelihood	−5324.99	−5346.52 to −5304.89	1555.90
	Rate ^e^	6.95 × 10^−3^	5.85 × 10^−3^ to 8.13 × 10^−3^	3372.60
	tMRCA ^f^	6.334	5.508 to 7.269	5915.50
		**7 June 2017**		

^a^ See Supplementary Material Table S1 for strains included in this analysis. ^b^ In all cases, the mean values are shown. ^c^ HPD, high probability density values. ^d^ ESS, effective sample size. ^e^ Rate is shown in substitutions/site/year. ^f^ tMRCA, time of the most common recent ancestor, is shown in years. The date estimated for the tMRCA is indicated in bold.

## Data Availability

All the data is provided within the article or in the supplementary material provided.

## Supplementary Materials

The following supporting information can be downloaded at: https://www.mdpi.com/article/10.3390/v17070924/s1, Figure S1: 3D Model of the Receptor Binding Site of Recommended Vaccine Strain A/chicken/Ghana/AVL763/2021 (Clade 2.3.4.4b); Table S1: Sequences from GISAID’s EpiFlu™ Database on which this research is based.
