# Supplementary material for: Phylogenetic and Mutation Analysis of Hemagglutinin Gene from Highly Pathogenic Avian Influenza Virus H5 Clade 2.3.4.4b in South America
Source: Viruses. 2025 Jun 28;17(7):924. doi: 10.3390/v17070924 (PMC12298084; doi:10.3390/v17070924)

Supplementary Figure S1. 3D Model of the Receptor Binding Site of Recommended Vaccine Strain A/chicken/Ghana/AVL763/2021 (Clade 2.3.4.4b) (Downloaded from https://www.rcsb.org/3d-view).

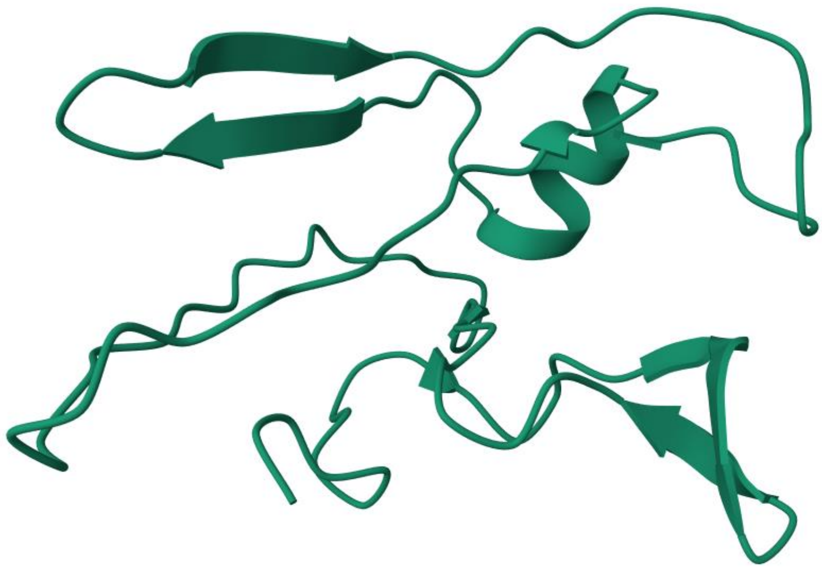

Supplement: Supplementary file 1 [file viruses-17-00924-s001.zip › viruses-3562860-Supplementary Figure.pdf]
